# Rapid intrapartum test for maternal group B streptococcal colonisation and its effect on antibiotic use in labouring women with risk factors for early-onset neonatal infection (GBS2): cluster randomised trial with nested test accuracy study

**DOI:** 10.1186/s12916-021-02202-2

**Published:** 2022-01-14

**Authors:** Jane P. Daniels, Emily Dixon, Alicia Gill, Jon Bishop, Mark Wilks, Michael Millar, Jim Gray, Tracy E. Roberts, Jane Plumb, Jonathan J. Deeks, Karla Hemming, Khalid S. Khan, Shakila Thangaratinam, on behalf of the GBS2 Collaborative Group, Khaled Ahmed, Julie Dodds, Maria D’Amico, Kostas Tryposkiadis, Angela Whiley, Patrick Moore, Ruvimbo Lorraine Munetsi, Pallavi Karkhanis, Anne Deans, Sanjula Sharma, Gemma Wright, Manjula Subramanian, Irene Ray, Dibyenda Datta, Lauren Lacey, Johnathon Pepper, Ruth Mason, Neil Shah, Katharina Anwar, Neena Navaneetham, Shad Husain, Phillip Bennett, Geraldine Masson, Hristina Raykova, Matthew Hogg, Bashir Dawalatly, Lakshmi Thirumalaikumar, Kate Townsend, Gerry Collins, Paul Heath, Kerry Hood, Stavros Petrou, Ben Stenson, Sarah McMullen, Julia Saunders, Alison Stanley, Stephen Walters, Patrick Bossuyt, Ruth Gilbert, Rhona Hughes

**Affiliations:** 1 Nottingham Clinical Trials Unit, School of Medicine, University of Nottingham, Nottingham, NG7 2RD UK; 2 Birmingham Clinical Trials Unit, College of Medical and Dental Sciences, University of Birmingham, Birmingham, UK; 3 Barts Health NHS Trust, London, UK; 4 Queen Mary University of London, London, UK; 5 Birmingham Women’s & Children’s NHS Foundation Trust, Birmingham, UK; 6 Institute of Applied Health Research, College of Medical and Dental Sciences, University of Birmingham, Birmingham, UK; 7 Group B Strep Support, JYW House, Bridge Road, Haywards Heath, UK; 8 NIHR Birmingham Biomedical Research Centre, University Hospitals Birmingham NHS Foundation Trust and University of Birmingham, Birmingham, UK; 9 Department of Preventive Medicine and Public Health, University of Granada, Granada, Spain; 10 Institute of Metabolism and System Research, College of Medical & Dental Sciences, University of Birmingham, Birmingham, UK

**Keywords:** Group B *Streptococcus*, Colonisation, Randomised controlled trial, Accuracy, Labour, Pregnancy, Antibiotics

## Abstract

**Background:**

Mother-to-baby transmission of group B *Streptococcus* (GBS) is the main cause of early-onset infection. We evaluated whether, in women with clinical risk factors for early neonatal infection, the use of point-of-care rapid intrapartum test to detect maternal GBS colonisation reduces maternal antibiotic exposure compared with usual care, where antibiotics are administered due to those risk factors. We assessed the accuracy of the rapid test in diagnosing maternal GBS colonisation, against the reference standard of selective enrichment culture.

**Methods:**

We undertook a parallel-group cluster randomised trial, with nested test accuracy study and microbiological sub-study. UK maternity units were randomised to a strategy of rapid test (GeneXpert GBS system, Cepheid) or usual care. Within units assigned to rapid testing, vaginal-rectal swabs were taken from women with risk factors for vertical GBS transmission in established term labour. The trial primary outcome was the proportion of women receiving intrapartum antibiotics to prevent neonatal early-onset GBS infection. The accuracy of the rapid test was compared against the standard of selective enrichment culture in diagnosing maternal GBS colonisation. Antibiotic resistance profiles were determined in paired maternal and infant samples.

**Results:**

Twenty-two maternity units were randomised and 20 were recruited. A total of 722 mothers (749 babies) participated in rapid test units; 906 mothers (951 babies) were in usual care units. There was no evidence of a difference in the rates of intrapartum antibiotic prophylaxis (relative risk 1.16, 95% CI 0.83 to 1.64) between the rapid test (41%, 297/716) and usual care (36%, 328/906) units. No serious adverse events were reported. The sensitivity and specificity measures of the rapid test were 86% (95% CI 81 to 91%) and 89% (95% CI 85 to 92%), respectively. Babies born to mothers who carried antibiotic-resistant *Escherichia coli* were more likely to be colonised with antibiotic-resistant strains than those born to mothers with antibiotic-susceptible *E. coli*.

**Conclusion:**

The use of intrapartum rapid test to diagnose maternal GBS colonisation did not reduce the rates of antibiotics administered for preventing neonatal early-onset GBS infection than usual care, although with considerable uncertainty. The accuracy of the rapid test is within acceptable limits.

**Trial registration:**

ISRCTN74746075. Prospectively registered on 16 April 2015

**Supplementary Information:**

The online version contains supplementary material available at 10.1186/s12916-021-02202-2.

## Background

Group B streptococcus (GBS) is a Gram-positive pathogen found in the gut and genital tract of one in five women; a third of these women pass the bacteria to their baby during pregnancy or labour [1]. Most babies colonised with GBS are asymptomatic. GBS is one of the leading causes of early-onset neonatal infection, with a global pooled incidence rate of 0.49 per 1000 live births (95% confidence interval [CI] 0.43 to 0.56) and disproportionately high mortality and morbidity [2, 3]. In the UK and Ireland, at least two babies are diagnosed every day with GBS infection (at 0–90 days); one baby dies every week, and one survives with long-term disability [2].

In order to prevent neonatal early-onset GBS infection, pregnant women colonised with GBS are offered antibiotics intrapartum, ideally at least 4 h before childbirth [4, 5]. Many countries, such as the USA, have national screening programmes that use culture-based tests to identify women colonised with GBS in late pregnancy [6]. But culture-based tests at 35–37 weeks of gestation have limited accuracy in predicting the maternal GBS colonisation status in labour [7, 8], take up to 48 h to produce a result, and are likely to be missed in women who go into labour preterm.

Some high-income countries, including the UK, remain uncertain about the balance between the benefits and harms of universal screening [9], and instead rely on a risk-based approach, where all women with risk factors are offered intrapartum antibiotic prophylaxis to prevent early-onset GBS infection in their babies [10]. Neonates are closely monitored for signs of infection and administered antibiotics if there is a diagnosis or suspicion of sepsis [11]. Since only a third of women with risk factors are colonised with GBS, a large proportion of women and their babies are unnecessarily exposed to antibiotics [12]. Rapid and accurate determination of the GBS colonisation status of the mother in labour can enable targeted administration of antibiotics in a timely fashion and reduce needless exposure to antibiotics in mothers whose babies maybe considered to be at risk of infection, but where the labouring mother is not actually colonised.

## Methods

### Study aims and designs

We undertook a cluster randomised trial to determine if the use of point-of-care intrapartum rapid test for maternal GBS colonisation, implemented at a maternity unit level, can reduce maternal and neonatal antibiotic exposure, compared with usual care where antibiotics are offered based on maternal risk factors. Testing of mothers without risk factors for early-onset infection in their babies was outside the commissioned scope of the trial.

We also assessed the real-time accuracy of a rapid nucleic acid amplification test to detect maternal GBS colonisation in women presenting to a labour ward with risk factors for neonatal early-onset GBS infection against a reference standard of selective enrichment culture in a nested test accuracy study.

In a subset of mother-child pairs, we determined the antibiotic resistance profile of any GBS isolated, and the carriage rate of other antibiotic-resistant bacteria and resistance genes in the maternal rectovaginal samples and compared the findings with the offspring’s faecal sample at 6–12 weeks of age.

### Study setting and participants

Twenty UK maternity units were clusters. The units were eligible to participate if they were prepared to accept a policy of rapid test-directed intrapartum antibiotic prophylaxis to prevent neonatal early-onset GBS infection and had access to microbiology facilities for selective enrichment bacteriological culture of GBS. Clinical midwives and doctors identified potential participants and screened for eligibility in various locations including the delivery suite, the maternity triage unit or the induction ward. No research-specific consent was obtained for the cluster trial and diagnostic study, although it was for the microbiology sub-study. Women in the rapid test units received information about the test and provided verbal assent to have the vaginal-rectal swab. Pregnant women were eligible for inclusion in the trial if they had one or more of the following risk factors: a previous baby with early- or late-onset GBS disease; GBS bacteriuria during the current pregnancy (irrespective of whether the GBS bacteriuria was treated at the time of diagnosis with antibiotics); GBS maternal colonisation of the vagina and/or rectum in the current pregnancy; suspected, diagnosed or established preterm labour (less than 37 weeks’ gestation); and maternal pyrexia (≥ 38 °C). Women were ineligible if they were under 16 years of age, less than 24 weeks’ gestation, were in the second stage of labour at admission or considered likely to give birth to their baby imminently, having a planned elective caesarean birth or their baby was known to have died in utero or had a congenital anomaly incompatible with survival at birth.

### Cluster randomised trial

#### Randomisation and masking

Randomisation of clusters was performed at the Birmingham Clinical Trials Unit using a minimisation algorithm incorporating the following factors: region (the Midlands, London and South East England), pre-trial intrapartum antibiotic usage rate (above or below the median of all sites) and the number of vaginal or emergency Caesarean births (above or below the median). Due to the differences in the strategies for testing women and for directing intrapartum antibiotic prophylaxis, it was not possible to blind women or their care team to the randomised allocation of their maternity unit.

#### Procedures in rapid test and usual care units

Maternity units randomised to the rapid test received a GeneXpert® Dx IV GBS rapid testing system (Cepheid, Sunnyvale, USA) and a supply of XpertGBS test cartridges. Trained clinical midwives obtained vaginal and rectal maternal samples using a double-headed swab. One swab was used immediately for the rapid test according to the manufacturer’s instructions, and a result was obtained in less than 55 min. If the rapid test had not been initiated on the GeneXpert machine within 15 min of taking the swab, the test was considered invalid. The other swab was used for the diagnostic test accuracy study.

The rapid test units were advised to go against national guidelines, and only women who tested positive for GBS with the rapid test or for whom a test result was not available were to be offered intrapartum antibiotic prophylaxis, unless there was a clinical reason for prescribing antibiotics, or if the woman requested antibiotics.

If the woman had not given birth 48 h after the test result was available, the test result was regarded as invalid, and it was advised that the woman should be re-swabbed and retested for GBS colonisation.

Usual care units followed their standard risk-based screening strategy where intrapartum antibiotic prophylaxis was offered to all women with risk factors.

The recommended antibiotic regimen for preventing early-onset neonatal GBS infection in both types of units in the study was in line with national recommendations, where benzyl penicillin is the first-choice antibiotic for GBS prophylaxsis [10]. Subsequent clinical management of mother and baby was based on local guidance [11].

#### Outcome measures

The primary outcome was the proportion of women with risk factors who received intrapartum antibiotic prophylaxis to prevent neonatal early-onset GBS infection. The secondary maternal outcomes were intrapartum maternal antibiotic administration for any indication, indications other than Caesarean section, any postpartum maternal antibiotic use and exposure to antibiotics for greater than 2 or 4 h before delivery. The neonatal outcomes were the proportion of newborns who receive antibiotics for any indication, with suspected or diagnosed early-onset sepsis requiring antibiotics and neonatal mortality at any time until discharge from the hospital. We also reported serious adverse events in the mother or newborn.

### Sample size

The proportion of women with risk factors for early-onset GBS infection in their newborns receiving intrapartum antibiotic prophylaxis was expected to be between 50 and 75%, from previous estimates and expected improvements in adherence to guidelines since then [12]. With a sample size per unit of 83 women and a minimum of 20 units, we expected the trial to have 90% power to detect a reduction to 63% in rapid test units (for a comparative control of 75%), assuming an intracluster coefficient of 0.01 [13]. This equated to a total sample size of approximately 664 per strategy group, rounded up to 1340 in total, for the cluster randomised trial.

### Statistical analysis

All trial analyses were conducted by intention-to-treat analyses according to the randomised allocation of the maternity unit, excluding any units who withdrew before data collection started and participants later found to be ineligible. For participant and cluster characteristics, we summarised the categorical data by frequencies and percentages. We summarised the continuous data by the number of responses, mean and standard deviation if deemed to be normally distributed and by the number of responses, median and interquartile range if the data appeared skewed. For the primary analysis in the cluster randomised trial, we used a mixed effects binomial regression with a log-link to estimate the relative risk, and a binomial model with an identity link to estimate the risk difference. Both models allowed for clustering by maternity unit as a random effect and adjusted for minimisation variables as fixed effects. If the binomial model with the identity link did not converge, we only reported the relative risk. In the case of non-convergence of the binomial model with a log-link, a Poisson model with robust standard errors was fitted. We used Kenward and Roger method to correct the potential inflation of the type I error rate due to the small number of clusters [14]. We used GLIMMIX in SAS to estimate model parameters, using a restricted pseudo-likelihood approach based on a marginal expansion which can be viewed as a generalised form of REML (“RMPL” option in GLIMMIX). A post hoc analysis tested sensitivity to the estimation procedure and small sample correction method by comparing results obtained from an adaptive quadrature method (with between-within small sample correction) and using a between-within correction with a restricted pseudo-likelihood marginal expansion approach (again with a between-within small sample correction) [15]. Overall inferences did not change. Where covariate adjustment was not practical, unadjusted estimates were produced and explained (e.g. not possible due to low event rate, lack of model convergence or poor recording accuracy of covariates).

We pre-specified subgroup analyses for the effects of the rapid test on the primary outcome in each of the categories based on the following maternal risk factors: maternal temperature of 38 °C or above observed whilst in labour, previous baby with GBS disease, GBS detected in current pregnancy and preterm labour (< 37 weeks’ gestation). We summarised the treatment effects within each sub-group separately and performed an interaction test between each subgroup variable and the test strategy allocation.

### Diagnostic test accuracy study

We compared the rapid test findings against the reference standard of selective enrichment culture in a cohort study nested within the randomised trial. The second vaginal-rectal swab collected for the trial was returned to the transport tube and sent to the local microbiology laboratory for selective enrichment culture to detect GBS according to the recommended methods [16]. For eligible women, a single swab taken from her baby’s ear canal was also processed in the local microbiology laboratory by selective enrichment culture to detect the presence of GBS. The rapid test results preceded those of the culture test, which was interpreted blindly to the rapid test. The main test accuracy outcomes were the sensitivity and specificity of the rapid test, but we also estimated neonatal and mother-to-baby GBS transmission rates.

#### Sample size

For the test to be proven useful, it should detect a higher proportion of maternal GBS colonisations than other tests, but not at the cost of low specify or overdiagnosis. In the GBS1 study, intrapartum antibiotic prophylaxis directed by screening with enriched culture at 35–37 weeks’ gestation was considered to be the most acceptable cost-effective strategy [12], where the sensitivity of the antenatal screening test was 75.8% (95% CI 47.2 to 91.5%) [17]. If the sensitivity of the proposed rapid test was higher than 90%, which was the approximate upper limit of the enriched culture test sensitivity, we expected the rapid test performance to be acceptable for use in clinical practice. A sample of 676 women would have 90% power to show that the estimated sensitivity of the rapid test was greater than a fixed value of 90%, based on 167 cases of maternal GBS colonisation in the units randomised to use the rapid test and 10% failed tests.

#### Statistical analysis

We estimated the diagnostic accuracy of the rapid test through the standard calculations of sensitivity and specificity. Point estimates were presented with 95% confidence intervals that were calculated using binomial exact methods [18]. We also undertook a binomial proportion test to compare the observed sensitivity with a hypothesised minimal performance value of 90%.

### Microbiological study of bacterial antibiotic resistance

In a subset of women from sites in London and South East England that were randomised to the rapid test strategy, we obtained individual consent to test additional vaginal-rectal maternal swabs and the faecal samples of their babies from 6 to 12 weeks of age for maternal antibiotic resistance profile of GBS, maternal colonisation by other antibiotic-resistant bacteria and the carriage of those specific bacteria or resistance elements by the infant. The swabs underwent selective enrichment culture for GBS, methicillin-resistant *Staphylococcus aureus* (MRSA), vancomycin-resistant enterococci (VRE) and extended-spectrum β-lactamase producing (ESβL) *Enterobacteriaceae*. The presence of any bacteria of interest was profiled by a variety of techniques (including, but not necessarily limited to, antibiotic resistance, molecular/genetic characterisation and matrix-assisted laser desorption ionisation time of flight mass spectrometry). Antibiotic resistance was tested using the EUCAST methods and break points [19]. *Enterobacterales* were tested for sensitivity to ampicillin, piperacillin and tazobactam, amoxicillin and clavulanate, cefpodoxime, gentamicin, cefuroxime, amikacin, co-trimoxazole, temocillin, ceftazidime, ertapenem and ciprofloxacin on Muller Hinton agar. Molecular testing for Gram-negative antibiotic resistance genes was performed using the GSL EasyScreen™ Sample Processing Kit (SP006, Genetic Signatures Ltd., Newtown, Australia) designed to rapidly isolate nucleic acids (DNA and RNA) from clinical samples via an automated purification system. Fisher’s exact test was used to compare the proportions. We reported the relative risk of carriage of resistant *E. coli* or resistance genes in infants born to mothers with or without carriage of strains with specific characteristics using a binomial regression model with a log-link.

## Results

**Fig. 1:**
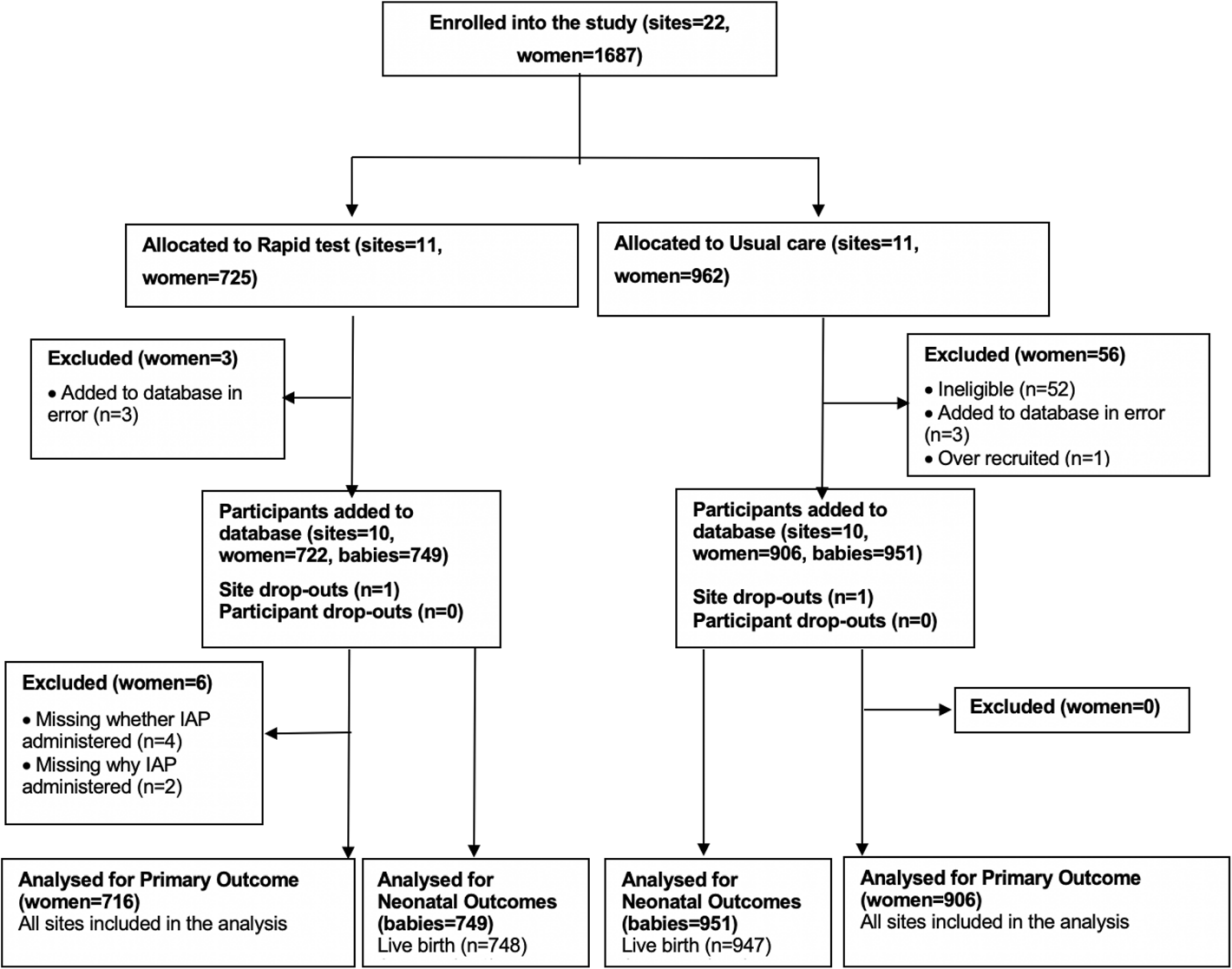
Site and participant flow through GBS2 study

Overall, we randomised 22 maternity units, of which 20 units contributed participants to the cluster randomised trial. Two sites withdrew after the randomisation and before the accrual of any data. One site that was allocated to usual care withdrew as it lost a key staff member, and the other, allocated to rapid test, then decided it could not implement the no-consent model, despite a waiver from the ethics committee. Of the 1628 eligible participants included in the dataset, 722 were in the rapid test and 906 in usual care units and gave birth between March 2018 and April 2019. There were 67 pairs of twins and 3 trios of triplets, resulting in 749 neonates in the rapid test units and 951 in the usual care units. We stopped recruitment when the sample size requirements were met. Figure 1 provides the details of the flow of participants in the trial.

### Characteristics of included clusters and participants

There were ten clusters each from London, South East England and Midlands; in each region, five maternity units were randomised to the rapid test or usual care strategies. The participants were similar in age, parity and mode of delivery (Table 1).

**Table 1:** Characteristics of clusters and participants in the GBS2 trial

Cluster	Characteristics	Rapid test (***n*** = 10)	Usual care (***n*** = 10)	Overall (***n*** = 20)
Region	London and South East England	5 (50%)	5 (50%)	10 (50%)
	Midlands	5 (50%)	5 (50%)	10 (50%)
No. of vaginal deliveries or emergency Caesarean births	Median [IQR]	4539 [3567–5583]	3996 [2930–5050]	4218 [2942–5168]
Estimated IAP rate amongst all vaginal deliveries	Median [IQR]	25.3 [22.8–32.6]	27.5 [9.9–31.7]	26.4 [13.4–32.2]
**Participant**	**Characteristics**	**Rapid test (*n*** **= 722)**	**Usual care (*n*** **= 906)**	**Overall (*n*** **= 1628)**
Region	London and South East England	375 (52%)	458 (51%)	833 (51%)
	Midlands	347 (48%)	448 (49%)	795 (49%)
Age, years	Mean (*SD*)	29.3 (5.8)	30.1 (5.8)	29.7 (5.8)
	Missing	1	0	1
Onset of labour	Spontaneous	343 (48%)	527 (58%)	870 (53%)
	Induced	354 (49%)	364 (40%)	718 (44%)
	Missing	25 (3%)	15 (2%)	40 (2%)
Type of delivery	Spontaneous vaginal	439 (61%)	542 (60%)	981 (60%)
	Instrumental	102 (14%)	131 (14%)	233 (14%)
	Emergency Caesarean	173 (24%)	233 (26%)	406 (25%)
	Missing	8 (1%)	0 (0%)	8 (< 1%)
Multiparity	Yes	465 (64%)	585 (65%)	1050 (65%)
	No	255 (35%)	321 (35%)	576 (35%)
	Missing	2 (< 1%)	0 (0%)	2 (< 1%)
Maternal risk factor for neonatal GBS infection	One risk factor	674 (93%)	841 (93%)	1515 (93%)
	Maternal temperature ≥ 38 °C	55 (8%)	139 (15%)	194 (12%)
	Previous baby with GBS	35 (5%)	40 (4%)	75 (5%)
	GBS in this pregnancy	293 (41%)	278 (31%)	571 (35%)
	Preterm labour	291 (40%)	384 (42%)	675 (41%)
	Two risk factors	46 (6%)	63 (7%)	109 (7%)
	Three risk factors	2 (< 1%)	2 (< 1%)	4 (< 1%)

*IAP* intrapartum antibiotic prophylaxis, *IQR* interquartile range, *SD* standard deviation

The proportion of women with risk factors such as preterm labour and previous baby with GBS was similar. There was a higher proportion of women with a known diagnosis of GBS colonisation in current pregnancy (41%) in the rapid test units than the usual care (31%) units; more women had intrapartum pyrexia in the usual care units (15%) than in the rapid test units (8%).

### Implementation of rapid test

At the cluster level, we achieved complete compliance with all sites allocated to the intervention using the rapid test technology. In the 241 women who were rapid test positive, 78% (190/241) received intrapartum antibiotic prophylaxis to prevent neonatal GBS infection and 8% (20/241) received antibiotics for other reasons; 13% of women (31/241) who tested positive did not receive any antibiotics. Of the 316 women who were rapid test negative, 56% (176/316) received antibiotics. Of these, 17% were for prevention of neonatal GBS infection (52/316), 15% for maternal pyrexia (48/316), 12% prior to Caesarean section (12/316), 6% for maternal request (19/316) and 18% for other reasons (57/316). Supplementary Table S1 shows the adherence to the test result and Supplementary Table S2 the various indications for antibiotics administration.

### Effects of rapid intrapartum test on maternal and neonatal exposure to antibiotics

There were no statistically significant differences in the proportion of women receiving intrapartum antibiotic prophylaxis for preventing GBS infection in the newborn (adjusted relative risk aRR 1.16, 95% CI 0.83 to 1.64) between the rapid test (41%, 297/716) and the usual care (36%, 328/906) units (Table 2). The overall rates of intrapartum antibiotic prophylaxis administered to the mother for any reason were also similar between the two strategies (aRR 0.99, 95% CI 0.81 to 1.21). Women in the rapid test units were more likely to have received antibiotics at least 4 h before childbirth than those in the usual care units (adjusted risk difference aRD 0.16; 95% CI 0.06 to 0.27) Supplementary Table S3.

**Table 2:** Effects of a rapid test for GBS on maternal exposure to antibiotics compared to usual care

Outcome	Rapid test, ***n*** (%)	Usual care, ***n*** (%)	Adjusted risk difference (95% CI)^**1**^	Adjusted relative risk (95% CI)^**2**^
*Maternal primary*	**(*n*** **= 722)**	**(*n*** **= 906)**		
IAP for GBS				
Yes	297 (41%)	328 (36%)	0.05 (− 0.07, 0.18)	1.16 (0.83, 1.64)
No	419 (59%)	578 (64%)		
Missing	6	0		
*Maternal secondary*				
IAP for any indication				
Yes	484 (67%)	602 (66%)	− 0.007 (− 0.14, 0.12)	0.99 (0.81, 1.21)
No	238 (33%)	307 (34%)		
Missing	4	0		

*IAP* intrapartum antibiotic prophylaxis

^1^Risk difference; estimates < 0 favour rapid test

^2^Relative risk; estimates < 1 favour rapid test. Analyses adjusted for cluster size, unit birth rate, and estimated pre-trial IAP rates

Subgroup analysis by individual maternal risk factors did not show any statistically significant differences between the two strategies in the rates of intrapartum antibiotics administered to prevent neonatal GBS infection Supplementary Table S4. There were no reports of maternal anaphylaxis due to antibiotic administration or any reports of inoculation injury.

The neonates born to women in the rapid test units (33%, 244/749) had a significantly lower risk of receiving antibiotics for any reason by 29% (aRR 0.71, 95% CI 0.54 to 0.95) than those in the usual care units (44%, 412/951). There was a 37% reduction in the proportion of neonates given antibiotics for suspected early-onset sepsis (aRR 0.63, 95% CI 0.43 to 0.92) in the rapid test units (25%, 187/749) than usual care (39%, 374/951) units (Table 3). Supplementary Table S5 provides the various indications for the administration of antibiotics to the newborns in both types of units. In three-quarters of such neonates (72% rapid test, 73% usual care) commenced on antibiotics for suspected sepsis, the infection was subsequently ruled out and the antibiotics were discontinued (Supplementary Table S6). There were 3 neonatal deaths amongst 749 births in the rapid test units and 8 death in 951 births in the usual care units.

**Table 3:** Effects of a rapid test for GBS on neonatal exposure to antibiotics compared to usual care

Outcome	Rapid test, ***n*** (%)	Usual care, ***n*** (%)	Adjusted risk difference (95% CI)^**1**^	Adjusted relative risk (95% CI)^**2**^
*Neonatal (live births only)*	**(*n*** **= 748)**	**(*n*** **= 947)**		
Antibiotics for any indication				
Yes	244 (33%)	412 (44%)	− 0.13 (− 0.23, − 0.02)	0.71 (0.54, 0.95)
No	493 (67%)	534 (56%)		
Missing	11	1		
Antibiotics for suspected early-onset sepsis				
Yes	187 (25%)	374 (39%)	− 0.15 (− 0.26, − 0.04)	0.63 (0.43,0.92)
No	548 (75%)	572 (61%)		
Missing	13	1		
Neonatal mortality (all neonates)*	**(*n*** **= 749)**	**(*n*** **= 951)**		
Yes	3 (0.4%)	8 (0.84%)		0.48 (0.10, 2.21)
No	746 (99.6%)	943 (99.16%)		
Missing	0	0		

*Relative risk estimated from an unadjusted Poisson mixed effects model with robust standard errors, no model for the risk difference converged

^1^Risk difference; estimates < 0 favour rapid test

^2^Relative risk; estimates < 1 favour rapid test. Analyses adjusted for cluster size, unit birth rate, and estimated pre-trial IAP rates

### Accuracy of rapid test for diagnosing maternal GBS colonisation

**Fig. 2:**
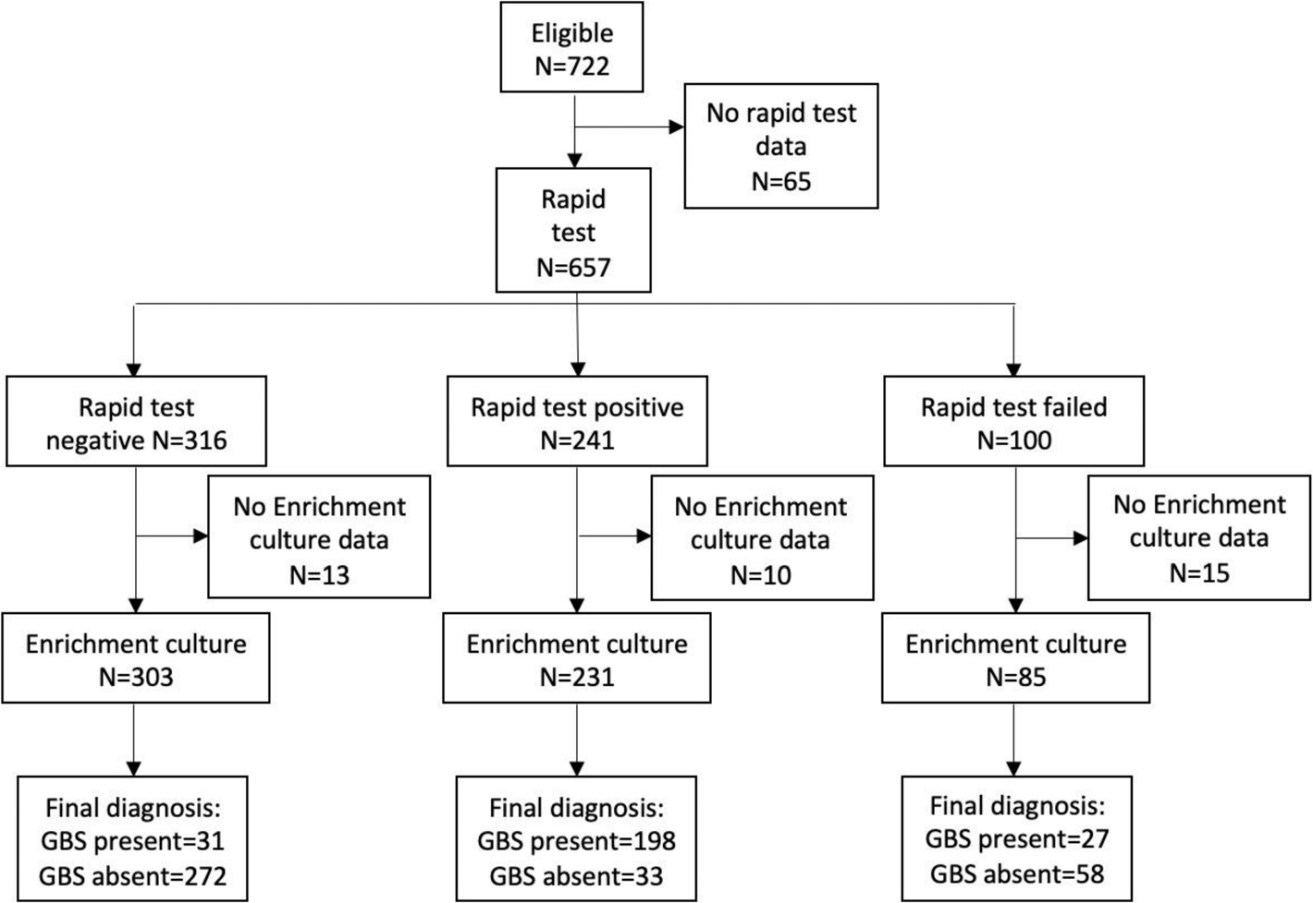
Flow chart of the test data from rapid test and selective enrichment culture

Overall, 557 of the 722 (77%) women in the rapid test units contributed results to the rapid intrapartum test (whether first or subsequent tests), 619 (86%) contributed to selective enrichment culture and 534 (74%) contributed information on both tests (Fig. 2). The sensitivity of the rapid test was 86% (95% CI 81–91%), and the specificity was 89% (95% CI 85–92%). The sensitivity of the rapid test was not statistically different from the target value of 90% (*p* = 0.052) (Table 4).

**Table 4:** Accuracy of rapid test in diagnosing maternal GBS colonisation status

		Selective enrichment culture		Sensitivity (95% CI)	Specificity (95% CI)
		Positive	Negative		
**Rapid test**	**Positive**	198 (86%)	33 (11%)	86% (81–91%)	89% (85–92%)
	**Negative**	31 (14%)	272 (89%)		

Sensitivity analyses that excluded women who were tested using only vaginal swabs and by excluding women who had vaginal cleansing or lubrication with an antimicrobial solution prior to the test yielded similar sensitivity and specificity estimates (Supplementary Table S7).

### Maternal and neonatal GBS colonisation

The overall prevalence of maternal GBS colonisation was 43% (95% CI 39 to 48%) by rapid test method and 41% (95% CI 37–45%) by selected enrichment culture (Supplementary Table S8). The overall rates of maternal GBS colonisation were similar when women who contributed a vaginal but not a rectal swab were excluded (Supplementary Table S8). The overall neonatal colonisation rate was 11% (49/445; 95% CI 8 to 14%) in the 445 babies assessed using selective enrichment culture of neonatal ear swabs. Neonatal colonisation was detected in one in five neonates born to women colonised with GBS as per the selective enrichment culture (19%, 35/186) or rapid test (21%, 38/184) (Table 5).

**Table 5:** Rates of neonatal GBS colonisation in babies born to women tested for GBS using rapid test or selective enrichment culture

Maternal GBS colonisation status		Neonatal colonisation present
Selective enrichment culture*	Positive	35/186 (19%)
	Negative	12/240 (5%)
Rapid test**	Positive	38/184 (21%)
	Negative	7/203 (3%)

*A total of 619 women had results for selective enrichment culture; 418 of these mothers had 426 neonates providing ear swabs

**A total of 557 women had results for rapid test; 380 of these mothers had 387 neonates providing ear swabs

### Microbiological and antibiotic resistance profiles

Overall, 117 women provided samples using vaginal-rectal swabs to determine the antibiotic resistance profile of GBS and other bacteria, with 64 paired samples (63 mothers and 64 infants including one set of twins); 60 paired samples were available for molecular testing (59 mothers, 60 infants).

We isolated GBS in 33% (39/117) maternal vaginal-rectal and in two infant faecal (2/64) samples; 82% (32/39) of maternal GBS isolates were tetracycline-resistant, 23% (9/39) erythromycin-resistant and 18% (7/39) were clindamycin-resistant. No penicillin-resistant maternal GBS isolates were identified. *E. coli* was isolated in 73% of the maternal samples (85/117). Half of all *E. coli* isolated were resistant to ampicillin (54%, 46/85), 44% (37/85) to amoxycillin/clavulanate, 25% (21/85) to trimethoprim/sulphamethozaxole, 6% (5/85) to ciprofloxacin, 5% (4/85) to gentamicin and 4% (3/85) to extended-spectrum β-lactamase (ESβL); 21% (18/85) were resistant to three or more antibiotic classes (multi-resistant MR). Vancomycin-resistant enterococci (VRE) were isolated from one infant and one unrelated maternal sample. There was no methicillin-resistant *Staphylococcus aureus* (MRSA) isolated from mother or infant samples. Gram-negative antibiotic resistance genes were identified in the 128 samples from mother-infant pairs: TEM, CTX-M, SHV, OXA-23,48,51(like), CMY, IMI, VIM, MCR-1, DHA, GES, KPC and NDM. Only TEM and CTX-M were present in 10 or more maternal samples; SHV (which is carried by *Klebsiella* species predominantly) was found in 14 of 60 infant samples compared with only 5 of 59 maternal samples, possibly reflecting differences in mother and infant *Klebsiella* colonisation (*p* < 0.05).

There was a significant association for colonisation status in faecal samples of infants at 6–12 weeks of age and maternal vaginal-rectal colonisation status intrapartum for co-trimoxazole resistant and multi-resistant *E. coli* in both unadjusted and adjusted estimates (Table 6). The limited sample size precluded the convergence of a “full” model adjusted for all variables, so no estimates of relative risks or confidence intervals taking account of all variables are presented.

**Table 6:** Relative risk of baby colonisation born to mothers colonised with antibiotic-resistant *Escherichia coli* and other resistance genes compared with non-colonised mothers

Outcome	Relative risk of colonisation in babies at 6–12 weeks of age by maternal colonisation status (***n*** = 64 babies)					
	Unadjusted estimate	Adjusted for mode of delivery	Adjusted for gestational age at birth	Adjusted for neonatal antibiotic use	Adjusted for maternal antibiotic use	Adjusted for neonatal ICU admission
***E. coli*** **detected**	1.3 (0.70, 2.47)	1.29 (0.68, 2.46)	1.35 (0.72, 2.52)	1.31 (0.70, 2.46)	1.32 (0.70, 2.49)	–
**Ampicillin-resistant** ***E. coli***	1.98 (0.94, 4.16)	2.29 (1.13 ,4.64)	2.13 (1.04, 4.39)	1.91 (0.92, 3.97)	2.00 (0.95, 4.23)	–
**Co-amoxiclav-resistant** ***E. coli***	1.93 (0.81, 4.58)	2.14 (0.88, 5.19)	2.24 (0.97, 5.19)	1.95 (0.84, 4.53)	1.84 (0.77, 4.40)	–
**Co-trimoxazole-resistant** ***E. coli***	4.82 (1.12, 20.8)	4.44 (0.97, 20.3)	5.16 (1.21, 22.0)	4.71 (1.04, 21.4)	4.51 (1.03, 19.7)	4.55 (1.05, 19.6)
**Multiple resistance in** ***E. coli***	6.50 (1.22, 34.7)	5.14 (0.99, 26.6)	6.70 (1.29, 34.8)	9.52 (1.93, 46.9)	6.39 (1.17, 34.8)	6.13 (1.15, 32.7)
**TEM resistance gene**	1.71 (0.96, 3.06)	1.83 (1.01, 3.33)	1.92 (1.07, 3.46)	1.89 (1.07, 3.34)	1.67 (0.91, 3.06)	1.87 (1.01, 3.46)
**CTX-M resistance gene**	2.00 (0.98, 4.06)	1.87 (0.88, 3.99)	1.88 (0.93, 3.79)	1.88 (0.94, 3.74)	1.92 (0.95, 3.89)	1.86 (0.92, 3.77)

Empty cells correspond to analyses that failed to converge and produce estimates. Estimates are obtained through log-binomial regression models in which the presence or absence of infant carriage is modelled by the presence or absence of maternal carriage alone and the value of the specified adjustment variable. The gestational age variable is continuous, and all other variables are binomial

## Discussion

In pregnant women with risk factors for early-onset GBS infection in their babies, the use of a point-of-care rapid test in labour to diagnose maternal GBS colonisation increased the administration of intrapartum antibiotics to prevent neonatal GBS infection by a small amount, compared with the usual care strategy of risk-factor based antibiotics administration, but with considerable uncertainty. The overall maternal exposure to antibiotics for any reason was not reduced with the use of the rapid test compared with usual care, whilst more women received an adequate duration of intrapartum antepartum prophylaxis in the rapid test units.

The absence of a reduction in intrapartum antibiotic prophylaxis through the implementation of a rapid testing strategy can be attributed to the following reasons. Firstly, although all women in usual care units should have been offered antibiotics if they had risk factors for neonatal early-onset GBS infection, this was only administered to 36% of women, which was lower than our expected estimate. This highlights the low adherence to the national guidelines, a situation that has changed little since a surveillance study in 2014–2015 where 44% of women with risk factors received IAP [20]. Secondly, despite the negative rapid test results for GBS colonisation, clinicians still administered antibiotics for neonatal infection prophylaxis to 17% of women. We did not explore the reasons why clinicians were motivated to offer antibiotics in this circumstance.

Maternal GBS colonisation status is considered to be a predictor for neonatal sepsis and has been incorporated in prediction models for neonatal sepsis [21, 22]. A strategy of rapid testing for maternal GBS colonisation in clinical practice appeared to reduce the neonatal exposure to antibiotics, with fewer newborns diagnosed with suspected early-onset sepsis requiring antibiotics. Our findings indicate that neonatologists take into consideration the results of the rapid test to make decisions on whether to start antibiotic treatment in the newborn, thereby reducing unnecessary exposure of the newborns to antibiotics.

The rapid intrapartum test showed good sensitivity and specificity in diagnosing maternal GBS colonisation. Our test accuracy estimates reflect the expected accuracy of the test when implemented in routine clinical practice by midwives, unlike previous studies where the tests were invariably handled by laboratory staff.

There is some evidence to suggest an association between multi-drug resistant gram-negative bacterial colonisation in the mother with similar colonisation in the newborn at 6 weeks of age.

### Strengths and limitations

Our randomised trial successfully engaged with multiple maternity units across different regions of the UK, trained healthcare professionals and implemented the rapid test in routine clinical practice. The maternal risk factors for neonatal early-onset GBS infection aligned with the national recommendations [10], and similar estimated rates of IAP in both strategy groups prior to the trial suggest consistent implementation of these recommendations. We recruited more than the required numbers of clusters, so that the study achieved a sufficient sample size, even when units dropped out prior to recruitment. The cluster randomisation design avoided the risk of bias, since the availability of the rapid testing facility would make it difficult for healthcare professionals not to offer the test, given the rapid access to results. We provided rapid test machines only to units allocated to that strategy. We did not record whether women changed their intended place of birth due to knowledge of the randomised strategy.

In addition to the primary outcome of intrapartum antibiotics for prevention of early-onset neonatal GBS infection, we also reported other relevant outcomes such as maternal and neonatal exposure to antibiotics given for any reason, and rates of suspected early-onset sepsis in newborns requiring antibiotics. We considered the sensitivity of our assumptions to a range of proportions in the usual care group and a range of ICC values which we believed to be quite conservative. The ICC calculated at the end of the trial at 0.06 (95% CI 0.03–0.12) was within our assumption used in the sample size calculation. Our analysis of the primary outcome allowed for clustering at a maternity unit level as a random effect, corrected for the small number of clusters. The embedded test accuracy study allowed us to simultaneously assess the real-time accuracy of the test in clinical practice, in addition to minimising research waste. We minimised the risk of bias by performing the rapid tests and selective enriched culture tests independently and evaluated blindly to each other. The double-headed swab ensured that the index and reference tests were undertaken on contemporaneous samples.

The study had limitations. It is possible that eligible women may have been missed in the rapid test units, as unlike usual care units where the data were collected from recorded notes, the offer of a rapid test to the woman depended on the availability and training of staff. We observed potential differential ascertainment of eligible participants between the two strategies, with more women with recorded antenatal GBS colonisation recruited in the rapid test units, and with intrapartum fever in the usual care units. It is possible that women with known prior GBS colonisation may have been more likely to be offered the rapid test.

We were not able to blind the women and the clinical staff due to obvious differences in testing strategies. However, ascertainment of the primary outcome was objective based on recorded administration of antibiotic use. Since the indications for antibiotic administration were ascertained from the clinical records in the usual care units, it is possible that some of the antibiotics administered for GBS prophylaxis may have been classified under a different indication. This is similar to the finding of the Royal College of Obstetricians and Gynaecologists’ audit that highlighted problems with interpreting the definition of intrapartum antibiotic prophylaxis and its indication [23].

Although we had fewer women with paired samples for the rapid test and selective enrichment culture than our projected sample size, the actual rate of maternal GBS colonisation was higher than anticipated. In a previous comparable study, 89% of rapid tests yielded a result, whereas we observed a lower yield of 77% in our study [24]. There is potential for spectrum bias, as the rapid test was only done in women with risk factors for early-onset GBS infection in the baby, although previous work had shown similar post-test probabilities for maternal colonisation in women with and without risk factors [12]. We did not collect information on maternal ethnicity. Our findings on the association between infant antibiotic resistance profile and maternal colonisation status were limited by the small numbers of paired samples.

### Comparison to other studies

To date, there have been no randomised trials evaluating the use of rapid GBS test in routine clinical practice compared to usual care. A prospective cohort in France that compared the performance of intrapartum GeneXpert test to antenatal microbiological screening for maternal GBS colonisation, reported that universal rapid intrapartum test had the potential to have an absolute risk reduction of 0.925%, equivalent to 108 more women needing to be tested in labour and provided with intrapartum prophylaxis to prevent a single case of early-onset GBS neonatal infection that would be missed by the culture-based testing at 35–37 weeks [24]. Compared with the French study where 89% of rapid tests yielded a result, we observed a lower yield of 77% in our study.

A meta-analysis of 15 studies on the accuracy of various PCR platforms to detect maternal GBS colonisation reported produced pooled sensitivity of 93.7% and specificity of 97.6% [25]. These estimates were higher than what was observed in the GBS2 study, although sensitivities within primary studies using the GeneXpert system ranged from 83.0 to 98.5%. Our findings indicate the sensitivity of the GeneXpert test is consistent with late third trimester selective enrichment culture in determining the colonisation status at birth [7]. In our study, the rates of GBS colonised babies born to mothers colonised with GBS was lower (20%) than the 36% reported in a previous meta-analysis of six small studies (308 women and 117 babies colonised) [1], which may be influenced by the treatment provided in our study. Although we did not assess the acceptability of the rapid test to women in this study, in our previous UK GBS rapid testing study, we found that 94% (984/1043) of mothers were happy or very happy with the way the swabs were taken, and 94% (975/1036) confident in its use in routine care [12].

## Conclusion

The use of a point-of-care rapid test in clinical practice to detect maternal GBS colonisation does not reduce the rate of antibiotics administered to the mother with risk factors to prevent neonatal early-onset GBS infection compared to the usual care policy of offering antibiotics based on only risk factors. The newborns appear to be less exposed to antibiotics with the rapid test than usual care. The GeneXpert rapid test has acceptable accuracy in detecting maternal GBS colonisation. Our sub-study highlights the potential for increased risk of multi-drug resistant *E. coli* in infants at 6–12 weeks of age in mothers colonised with such multi-drug resistant bacteria. The ongoing GBS3 (ISRCTN49639731) trial, which is comparing routine intrapartum rapid testing and antenatal microbiological testing in all women intending to deliver vaginally on the incidence of neonatal sepsis, will provide crucial information on the benefits of universal testing.

## Supplementary Information

**Additional file 1 **: **Table S1.** Antibiotic administration by GBS rapid test result. **Table S2.** Provision of antibiotic for different clinical reasons, by GBS test result. *Women can have more than one reason given for receiving intrapartum antibiotics. **Table S3.** Maternal antibiotic use in rapid test and usual care maternity units. *Unadjusted analysis due to lack of convergence with the adjusted model. **Estimated through an adjusted Poisson model with robust standard errors, estimates should be interpreted with caution as the covariance matrix for the random effects is not positive definite. **Table S4.** Use of GBS intrapartum antibiotic prophylaxis by risk factor subgroups. **Table S5.** Comparison of the reasons for neonatal antibiotic administration. *For 85 babies, there were >1 reason for receiving antibiotics; ^ For 129 babies, there were >1 reasons for receiving antibiotics; N is number of babies who received antibiotics and indication provided. **Table S6.** Management of babies administered IV antibiotics for suspected early neonatal sepsis. **Table S7.** Sensitivity analysis of accuracy of rapid test to diagnose GBS colonisation. **Table S8.** Prevalence of GBS maternal colonisation by test.

## Data Availability

We shall make data available to the scientific community with as few restrictions as feasible, whilst retaining exclusive use until the publication of major outputs. All data requests should be submitted to the corresponding author for consideration.

## Abbreviations

CI: Confidence interval
ESβL: Extended-spectrum β-lactamase producing *Enterobacterales*
GBS: Group B *Streptococcus*
IAP: Intrapartum antibiotic prophylaxis
ICC: Intra-cluster coefficient
*MRSA*: *Staphylococcus aureus*
*VRE*: Vancomycin-resistant enterococci
