# Supplementary material for: Rapid intrapartum test for maternal group B streptococcal colonisation and its effect on antibiotic use in labouring women with risk factors for early-onset neonatal infection (GBS2): cluster randomised trial with nested test accuracy study
Source: BMC Med. 2022 Jan 14;20:9. doi: 10.1186/s12916-021-02202-2 (PMC8759240; doi:10.1186/s12916-021-02202-2)
Supplement: Supplementary file 1 — Additional file 1 : Table S1. Antibiotic administration by GBS rapid test result. Table S2. Provision of antibiotic for different clinical reasons, by GBS test result. *Women can have more than one reason given for receiving intrapartum antibiotics. Table S3. Maternal antibiotic use in rapid test and usual care maternity units. *Unadjusted analysis due to lack of convergence with the adjusted model. **Estimated through an adjusted Poisson model with robust standard errors, estimates should be interpreted with caution as the covariance matrix for the random effects is not positive definite. Table S4. Use of GBS intrapartum antibiotic prophylaxis by risk factor subgroups. Table S5. Comparison of the reasons for neonatal antibiotic administration. *For 85 babies, there were >1 reason for receiving antibiotics; ^ For 129 babies, there were >1 reasons for receiving antibiotics; N is number of babies who received antibiotics and indication provided. Table S6. Management of babies administered IV antibiotics for suspected early neonatal sepsis. Table S7. Sensitivity analysis of accuracy of rapid test to diagnose GBS colonisation. Table S8. Prevalence of GBS maternal colonisation by test. [file 12916_2021_2202_MOESM1_ESM.docx]

# Supplementary File 1 – Additional Data

Table 1 - Antibiotic administration by GBS rapid test result

| **Rapid Test Result** | **Administration of Antibiotics** | | |  |  |
| --- | --- | --- | --- | --- | --- |
|  | Intrapartum antibiotics for GBS prophylaxsis | Antibiotic for other reasons | No antibiotics | Missing data | **Total** |
| Positive | 190 (78%) | 20 (8%) | **31 (13%)** | 0 (0%) | **241** |
| Negative | **52 (16%)** | 124 (39%) | 138 (44%) | 2 (1%) | **316** |
| Failed | 47 (47%) | 26 (26%) | 27 (27%) | 0 (0%) | **100** |
| Not performed | 7 (12%) | 15 (27%) | 33 (59%) | 1 (2%) | **56** |
| Missing test data | 1 (12%) | 2 (25%) | 5 (63%) | 0 (0%) | **8** |
| **Total** | **297** | **187** | **234** | **3** | **721** |

Table 2 - Provision of antibiotic for different clinical reasons, by GBS test result

| **Reasons for intrapartum antibiotic use^*^** | **Usual Care (n=906)** | **Rapid Test (n=721)** | | | | |
| --- | --- | --- | --- | --- | --- | --- |
|  |  | **Positive (n=241)** | **Negative (n=316)** | **Test failed or invalid (n=100)** | **Not performed (n=56)** | **Missing test data (n=8)** |
| GBS | 328 (52%) | 190 (30%) | 52 (8%) | 47 (7%) | 7 (1%) | 1 (0%) |
| Maternal pyrexia | 122 (62%) | 16 (8%) | 48 (24%) | 8 (4%) | 3 (2%) | 0 (0%) |
| Caesarean birth | 76 (52%) | 18 (12%) | 37 (25%) | 6 (4%) | 7 (5%) | 2 (1%) |
| Maternal request | 0 (0%) | 6 (22%) | 19 (70%) | 1 (4%) | 1 (4%) | 0 (0%) |
| Other reason | 120 (54%) | 8 (4%) | 57 (26%) | 29 (13%) | 6 (3%) | 2 (1%) |
| No antibiotics | 304 (57%) | 31 (6%) | 138 (26%) | 27 (5%) | 33 (6%) | 5 (1%) |

^*^Women can have more than one reason given for receiving intrapartum antibiotics.

Table 3 - Maternal antibiotic use in rapid test and usual care maternity units

| **Outcome** | **Rapid test**  **(n, (%))** | **Usual care**  **(n, (%))** | **Risk Difference (95% CI)** | **Relative Risk (95% CI)** |
| --- | --- | --- | --- | --- |
| **Intrapartum maternal antibiotic use for any indication other than caesarean section** | | | | |
| Yes | 454 (63%) | 559 (62%) | 0.007 (-0.11 to 0.13) | 1.01 (0.83 to 1.23) |
| No | 263 (37%) | 347 (38%) |  |  |
| TOTAL | 717 | 906 |  |  |
| Missing | 5 | 0 |  |  |
| **Postpartum maternal antibiotic use for any indication** | | | | |
| Yes | 146 (20%) | 203 (22%) | -0.02 (-0.12 to 0.08) | 0.92 (0.60 to 1.44), |
| No | 567 (80%) | 702 (78%) |  |  |
| TOTAL | 713 | 905 |  |  |
| Missing data | 9 | 1 |  |  |
| **IAP >2 hours before delivery** | | | | |
| Yes | 386 (79%) | 393 (71%) | 0.08 (-0.01 to 0.16)* | 1.16 (1.07 to 1.26)** |
| No | 104 (21%) | 157 (29%) |  |  |
| TOTAL | 490 | 550 |  |  |
| Missing time data | 12 | 76 |  |  |
| **IAP >4 hours before delivery** | |  |  |  |
| Yes | 332 (68%) | 299 (54%) | 0.16 (0.06 to 0.27) | 1.32 (1.15 to 1.52)** |
| No | 158 (32%) | 251 (46%) |  |  |
| TOTAL | 490 | 550 |  |  |
| Missing data | 12^3^ | 76^4^ |  |  |
|  |  |  |  |  |

*Unadjusted analysis due to lack of convergence with the adjusted model

**Estimated through an adjusted Poisson model with robust standard errors, estimates should be interpreted with caution as the covariance matrix for the random effects is not positive definite.

Table 4 - Use of GBS intrapartum antibiotic prophylaxis by risk factor subgroups

| **Risk factor** | **Rapid test group**  **[n, (%)]** | **Usual care group [n, (%)]** | **Risk Difference ^1^ (95% C.I.)** | **Relative Risk ^2^ (95% C.I.)** | **Interaction p-value** |
| --- | --- | --- | --- | --- | --- |
| **Maternal pyrexia (>38°C) in labour** | | | | | |
| Yes | 24/65 (37%) | 48/156 (31%) | 0.02 (-0.16, 0.20) | 1.09 (0.65, 1.83) | 0.79 |
| No | 273/651 (42%) | 280/750 (37%) | 0.05 (-0.07, 0.18) | 1.16 (0.82, 1.64) |  |
| TOTAL | 716 | 906 | - |  |  |
| **Previous baby with GBS disease** | | | | | |
| Yes | 17/51 (34%) | 23/53 (43%) | -0.04 (-0.26, 0.17) | 0.87 (0.48, 1.59) | 0.26 |
| No | 280/665 (42%) | 305/853 (36%) | 0.06 (-0.07, 0.19) | 1.19 (0.84, 1.67) |  |
| TOTAL | 716 | 906 |  |  |  |
| **GBS bacterium detected in current pregnancy** | | | | | |
| Yes | 156/330 (47%) | 130/331 (39%) | 0.09 (-0.04, 0.22) | 1.27 (0.89, 1.81) | 0.18 |
| No | 141/386 (37%) | 198/575 (34%) | 0.03 (-0.10, 0.15) | 1.06 (0.75, 1.50) |  |
| TOTAL | 716 | 906 |  |  |  |
| **Preterm labour** | | | | | |
| Yes | 121/320 (38%) | 152/432 (35%) | 0.03 (-0.10, 0.16) | 1.08 (0.76, 1.55) | 0.33 |
| No | 176/396 (44%) | 176/474 (37%) | 0.08 (-0.05, 0.21) | 1.23 (0.87, 1.76) |  |
| TOTAL | 716 | 906 |  |  |  |

Table 5 - Comparison of the reasons for neonatal antibiotic administration

| **Indications for IV antibiotic administration to the neonate** | **Rapid test (N= 749)*** | **Usual care (N= 951)^** | **Overall (N= 1700)** |
| --- | --- | --- | --- |
|  | [n, (% of all babies)] | | |
| Prophylactic antibiotics despite maternal IAP > or equal to 2 hours before delivery | 61 (8%) | 48 (5%) | 109 (6%) |
| Prophylactic antibiotics because maternal IAP < 2 hours before delivery | 21 (3%) | 29 (3%) | 50 (3%) |
| Prophylactic antibiotics because the mother did not receive any IAP | 17 (2%) | 15 (2%) | 32 (2%) |
| Suspected early onset neonatal sepsis | 187 (25%) | 374 (39%) | 561 (33%) |
| Other reason | 43 (6%) | 75 (8%) | 118 (7%) |

* For 85 babies, there were >1 reason for receiving antibiotics; ^ For 129 babies, there were >1 reasons for receiving antibiotics; N is number of babies who received antibiotics and indication provided.

Table 6 - Management of babies administered IV antibiotics for suspected early neonatal sepsis

| **Neonatal status** | **Rapid test (N=187)** | **Usual care (N=374)** | **Overall (N=561)** |
| --- | --- | --- | --- |
| Infection subsequently ruled out, clinically well and antibiotic treatment discontinued | 138 (72%) | 260 (73%) | 398 (72%) |
| Infection not microbiologically confirmed but a full course of antibiotics given | 46 (24%) | 80 (22%) | 126 (23%) |
| Infection caused by GBS | 3 (2%) | 8 (2%) | 11 (2%) |
| Infection caused by another bacterium | 6 (3%) | 10 (3%) | 16 (3%) |

Table 7 - Sensitivity analysis of accuracy of rapid test to diagnose GBS colonisation

|  | | **Selective enrichment culture** | |
| --- | --- | --- | --- |
|  |  | **Positive** | **Negative** |
| 1. **Excluding 90 women with only vaginal swabs** | | | |
|  |  | | |
| Rapid test | Positive | 177 (88%) | 25 (10%) |
|  | Negative | 24 (12%) | 221 (90%) |
| Accuracy parameters | Sensitivity | 88% (95% CI 84 - 93%), p=0.21 | |
|  | Specificity | 90% (95% CI 86 – 94%), p=0.50 | |
| 1. **Excluding 267 women with prior antimicrobial vaginal cleansing** | | | |
| Rapid test | Positive | 137 (89%) | 13 (10%) |
|  | Negative | 17 (11%) | 123 (90%) |
| Accuracy parameters | Sensitivity | 89% (95% CI 84 – 94%), p=0.37 | |
|  | Specificity | 89% (95CI% 86 – 93%), p=0.34 | |

Table 8 - Prevalence of GBS maternal colonisation by test

| **Swab results contributing to data** | **Test positive**  **n/N** | **Prevalence of GBS maternal colonisation**  **Rate (95% CI)** |
| --- | --- | --- |
| **All women with test data** | | |
| Rapid test | 241/657 | 43% (39-48%) |
| Selective enrichment culture | 256/619 | 41% (37-45%) |
| **Excluding 90 women contributing only vaginal swabs** | | |
| Rapid test | Rapid test | 45% (41-50%) |
| Selective enrichment culture | Selective enrichment culture | 44% (39-48%) |
